# Genomic strategies to understand causes of keratoconus

**DOI:** 10.1007/s00438-016-1283-z

**Published:** 2016-12-28

**Authors:** Justyna A. Karolak, Marzena Gajecka

**Affiliations:** 1 Department of Genetics and Pharmaceutical Microbiology, Poznan University of Medical Sciences, Swiecickiego 4, Poznan, 60-781 Poland; 2 Institute of Human Genetics, Polish Academy of Sciences, Strzeszynska 32, Poznan, 60-479 Poland

**Keywords:** Keratoconus, Complex disease, High-throughput methods, Next-generation sequencing, Candidate gene

## Abstract

Keratoconus (KTCN) is a degenerative disorder of the eye characterized by the conical shape and thinning of the cornea. The abnormal structure of KTCN-affected cornea results in loss of visual acuity. While many studies examine how environmental factors influence disease development, finding the genetic triggers has been a major emphasis of KTCN research. This paper focuses on genomic strategies that were implemented for finding candidate genes, including linkage and association studies, and presents different approaches of mutation screening. The advantages and limitations of particular tools are discussed based on literature and personal experience. Since etiology underlying KTCN is complex, numerous findings indicating heterogeneity of genetic factors involved KTCN etiology are presented.

## Introduction

Keratoconus (KTCN) is a degenerating, usually bilateral disorder of the eye characterized by progressive stromal thinning, which results in the conical shape of the cornea. These structural changes in the corneal layers induce optical aberrations, leading to a loss of visual acuity due to distorted blurred vision, which is caused by irregular astigmatism, and high myopia (Rabinowitz 1998). Although KTCN is sometimes referred to as a corneal dystrophy, it is not included in International Classification of Corneal Dystrophies (IC3D) (Weiss et al. 2008) and should be distinguished from this group of corneal diseases. However, co-occurrence of KTCN with many types of corneal dystrophies, including Avellino and Fuchs dystrophies (Igarashi et al. 2003; Salouti et al. 2010; Wilson et al. 2014), may indicate that common molecular mechanisms in the pathogenesis of these disorders are involved.

**Fig. 1:**
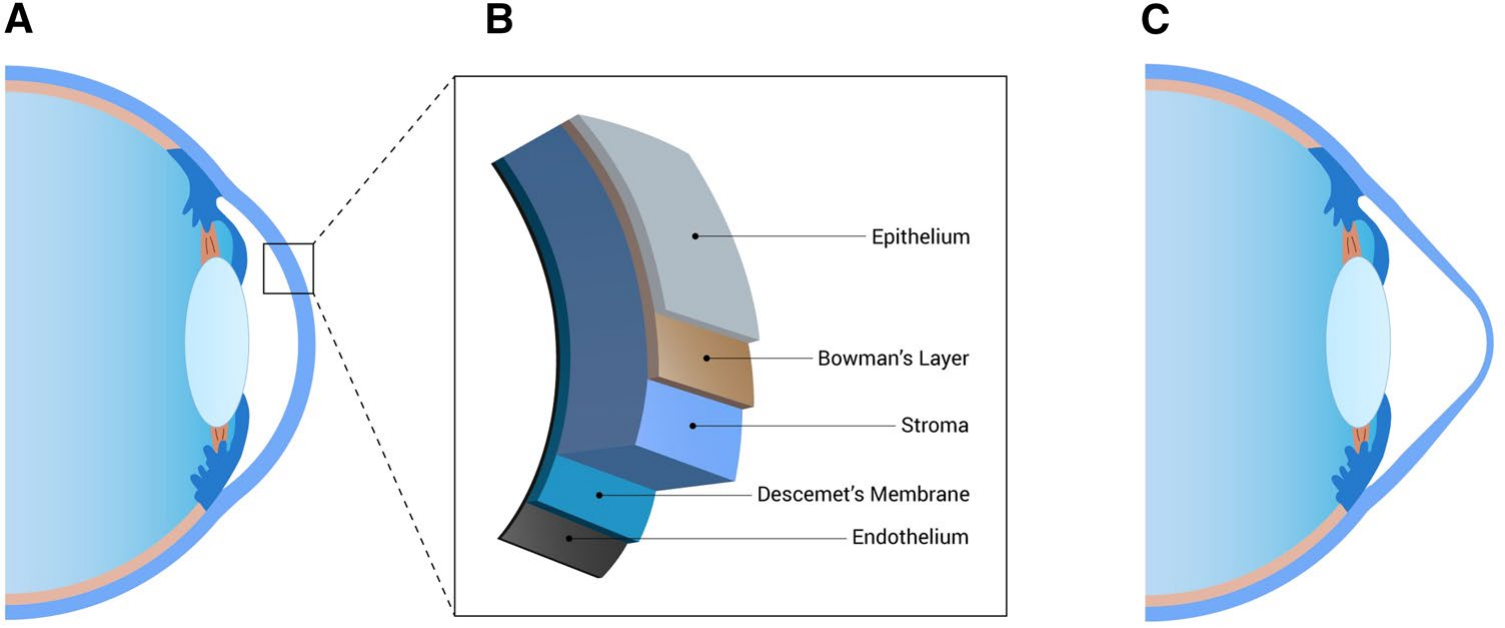
Schematic representation of the human eye. **a** Representation of healthy human eye with normal corneal thickness. **b** Structure of the human cornea. The cornea is a complex tissue comprised of five main layers (from the anterior to posterior margin): corneal epithelium, Bowman’s membrane, stroma, Descemet’s membrane, and endothelium. **c** Human eye with keratoconus with characteristic thinning and cone-like bulging of the cornea

Among the general population, the estimated frequency of KTCN is 1 in 2,000 individuals (Rabinowitz 1998), although up-to-date data are not available. The prevalence of KTCN may be different according to patient ethnic origin (Gokhale 2013). The reported prevalence of the disease may also vary depending upon the different diagnostic tests used in the particular studies. The early KTCN or *forme fruste* KTCN are not detectable at the slit lamp during the anterior segment examination, and in these cases, assessment of the corneal topographic pattern is required to obtain the accurate diagnosis (Saad and Gatinel 2010). The first symptoms of KTCN usually appear during the second decade or early in the third decade of life. The pathogenic features of KTCN may be observed in different layers of the cornea (Fig. 1) (Sherwin and Brookes 2004). These abnormalities include changes in morphology of epithelial cells (Sykakis et al. 2012), deposition of iron particles in the epithelial basement membrane, breaks in Bowman’s layer (Rabinowitz 1998), and thinning of stroma correlating with loss of collagen lamellae, altered collagen fibril orientation, and decreased keratocytes density (Patey et al. 1984; Meek et al. 2005; Mathew et al. 2011). Descemet’s membrane ruptures were recognized in KTCN cases with corneal hydrops (Rabinowitz 1998). The endothelium usually is not harmed in KTCN; however, sometimes, elongation and damage of endothelial cells are observed (Jeyabalan et al. 2013).

The clinical management of KTCN varies depending on the disease state and is focused on improvement of visual acuity and on the prevention of progression of the disease. Patients with the early KTCN may use spectacles or soft contact lenses. As the disease progresses, visual correction can be provided by rigid gas-permeable contact lenses, scleral lenses, or corneal collagen cross-linking. Some of the irregularities caused by mild-to-moderate KTCN may be also eliminated by insertion of intrastromal corneal ring segments. In advanced KTCN, due to progression of stromal thinning or scarring, corneal transplant surgery becomes necessary (Vazirani and Basu 2013). Corneal transplantation is required in up to 20% keratoconic eyes, thus KTCN is one of major causes for keratoplasty in developed countries (Faria-Correia et al. 2015).

Constant eye rubbing (McMonnies 2009), contact lens wear (Steahly 1978), atopy (Bawazeer et al. 2000), and UV light (Arnal et al. 2011) have been described as the major environmental and behavioral risk factors contributing to KTCN pathogenesis. Numerous genetic components, including familial inheritance (Naderan et al. 2016), a concordance between monozygotic twins in contrast to dizygotic twins (Tuft et al. 2012), and occurrence of syndromic KTCN (Elder 1994), indicate an evident genetic background of the disease. Whether KTCN is inherited according to Mendel’s laws or to a non-Mendelian pattern remains unknown. Recent segregation analyses suggest that KTCN is a complex trait, likely involving multiple genes, variable penetrance, and environmental contributions (Kriszt et al. 2014).

**Fig. 2:**
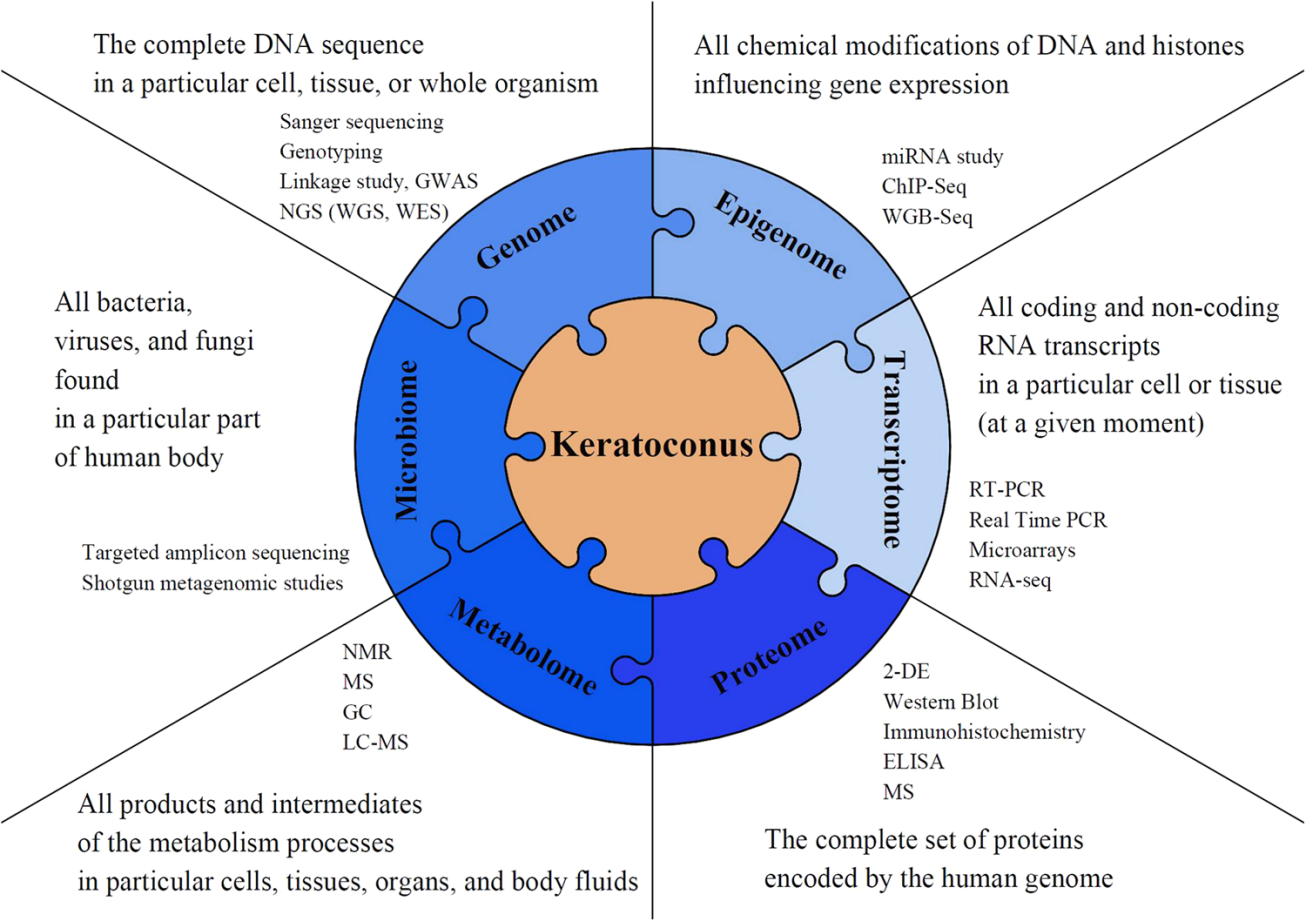
Integrating biological data from multiple approaches to understand keratoconus. To understand biological processes underlying keratoconus, integrated analysis of various biological aspects may be required. The figure summarizes information on numerous areas, including DNA sequence (genome), epigenetic modifications (epigenome), RNA transcripts (transcriptome), proteins (proteome), metabolites (metabolome), and microorganisms (microbiome). Each element in the array contains examples of techniques and technologies that can be used to study particular biological aspects. List of abbreviations: genome-wide association study (*GWAS*), next-generation sequencing (*NGS*), whole-genome sequencing (*WGS*), whole-exome sequencing (*WES*), reverse-transcription-PCR (*RT-PCR*), chromatin immunoprecipitation-sequencing (*ChIP-Seq*), RNA sequencing (*RNA-seq*), whole-genome bisulfite sequencing (*WGB-seq*), 2-D gel electrophoresis (*2-DE*), enzyme-linked immunosorbent assay (*ELISA*), nuclear magnetic resonance (*NMR*), mass spectrometry (*MS*), gas chromatography (*GC*), liquid chromatography–mass spectrometry (*LC–MS*)

Various tools may be adopted to understand etiology of KTCN (Fig. 2). This paper focuses on results obtained using different genetic approaches, including both simple molecular techniques and high-throughput technologies with computational and statistical methods. The advantages and limitations of particular techniques were discussed based on literature and personal experience.

## Linkage studies in KTCN

Linkage studies have been performed in large KTCN families with both affected and unaffected individuals. Linkage analyses have led to the identification of 20 chromosomal regions linked to KTCN (Table 1). KTCN loci have been mainly determined in families of Caucasian or Hispanic origin, with further loci recognized in other ethnicities or in the populations originated from different geographical regions, including Ecuadorian, Arabic, Caribbean African, Finnish, and Northwest Tasmanian. The loci mentioned above have been identified for both isolated KTCN (e.g., 3p14) and KTCN associated with other genomic diseases, such as Leber congenital amaurosis (e.g., 17p13), which is a clinically heterogeneous group of inherited childhood retinal degenerations (Hameed et al. 2000; Brancati et al. 2004).

**Table 1:** Chromosomal loci reported to show evidence of a linkage with keratoconus (data shown in chronological order)

Locus	Population	No. of families/inheritance	No. of individuals (no. of affected individuals)	No. of markers used for initial mapping	LOD	NPL	Positional candidate genes	Screened genes (method)	Detected mutations	References
17p13^a^	Pakistani	1/AR^b^	15 (4)	150 Microsatellite markers	3.21	–	*RETGC*-*1, PEDF*	*RETGC*-*1, PEDF* (Sanger sequencing)	No mutations	Hameed et al. (2000)
16q22.3-q23.1	Northern Finnish	20/AD^c^	76 (42)	292 Microsatellite markers	4.11	3.27	*LCAT, TAT, CHST6*	–	–	Tyynismaa et al. (2002)
20q12	Northwest Tasmanian	–	8 (8)	343 Dinucleotide repeat markers	–	–	*MMP9*	*MMP9* (cSNP analysis)	No mutations	Fullerton et al. (2002)
15q22.33-24.2^d^	Northern Irish	1/AD	30 (16)	350 Microsatellite markers	8.13	–	*CTSH, CRABP1, IREB2, RASGRF1*	*CTSH, CRABP1, IREB2, RASGRF1, ORF4L1, KIAA1055, ETFA, AWP1, REC14, KIAA1199, RCN2, FAH, IDH3A, MTHFS, ADAMTS7, MAN2C1, PTPN9, KIAA1024, ARNT2, BCL2A1, ISL2, C15ORF22* (*P24B*), *DNAJA4, FLJ14594, CIB2 (KIP2), C15ORF5, PSMA4, miR184* (Sanger sequencing)	*mir184* c.57C>T	Hughes et al. (2003), Dash et al. (2006), Hughes et al. (2011)
3p14-q13	Italian	1/AD	21 (10)	380 Markers	3.09	–	*COL8A1*	*COL8A1* (Sanger sequencing)	No mutations	Brancati et al. (2004), Aldave et al. (2007)
5q14.1-q21.1	Caucasian	1/AD	27 (14)	343 Microsatellite markers	3.48	–	*CAST, ARTS*-*1, PCSK1*	*CAST* (TaqMan genotyping)	*CAST* c.-40+7414T>C	Tang et al. (2005), Li et al. (2013b)
2p24	European, Arab, and Caribbean African	28/AD	216 (112)	382 Microsatellite markers	5.12	–	*OSR1, RhoB, GDF*-*7, FLJ21820, FLJ14249*	–	–	Hutchings et al. (2005)
2q	Hispanic	17/AD	93 (≥17 sib pairs)	380 Microsatellite markers	2.3	0.96	–	–	–	Li et al. (2006)
3p	Hispanic	17/AD	93 (≥17 sib pairs)	380 Microsatellite markers	2.2	1.69	–	–	–	Li et al. (2006)
4q	Caucasian/Hispanic	67/AD	351 (110 sib pairs)	380 Microsatellite markers	2.2	2.68	–	–	–	Li et al. (2006)
5q31	Caucasian/Hispanic	67/AD	351 (110 sib pairs)	380 Microsatellite markers	2.01	2.90	–	–	–	Li et al. (2006)
5p	Hispanic	17/AD	93 (≥17 sib pairs)	380 Microsatellite markers	2.5	2.64	–	–	–	Li et al. (2006)
9p	Hispanic	17/AD	93 (≥17 sib pairs)	380 Microsatellite markers	3.8	3.55	–	–	–	Li et al. (2006)
9q34	Caucasian/Hispanic	67/AD	351 (110 sib pairs)	380 Microsatellite markers	3.5	2.83	*GSN*	–	–	Li et al. (2006)
11p	Caucasian	40/AD	217 (≥40 sib pairs)	380 Microsatellite markers	2.3	1.98	–	–	–	Li et al. (2006)
12p	Caucasian/Hispanic	67/AD	351 (110 sib pairs)	380 Microsatellite markers	2.5	2.64	–	–	–	Li et al. (2006)
14q	Caucasian/Hispanic	67/AD	351 (110 sib pairs)	380 Microsatellite markers	2.6	2.23	–	–	–	Li et al. (2006)
17q	Hispanic	17/AD	93 (≥17 sib pairs)	380 Microsatellite markers	3.9	3.32	–	–	–	Li et al. (2006)
1p36.23-36.21	Australian	1/AD	11 (9)	10,000 SNP markers	1.94/3.4^e^	7.8^e^	*ENO1, CTNNBIP1, PLOD1, UBIAD1, SPSB1*	*ENO1, CTNNBIP1, PLOD1, UBIAD1, SPSB1* (Sanger sequencing)	No mutations	Burdon et al. (2008)
8q13.1-q21.11	Australian	1/AD	11 (9)	10,000 SNP markers	1.96/3.4^e^	7.8^e^	*TCEB1*	*TCEB1* (Sanger sequencing)	No mutations	Burdon et al. (2008)
5q21.2	Southern Italian	25/AD	136 (77)	382 Highly polymorphic markers	0.49	2.73	*LOX*	–	–	Bisceglia et al. (2009)
5q32-q33	Southern Italian	25/AD	136 (77)	382 Highly polymorphic markers	2.45	3.22	*SPARC*	–	–	Bisceglia et al. (2009)
9q21.13	Southern Italian	25/AD	136 (77)	382 Highly polymorphic markers	1.07	1.93	*ANXA1*	–	–	Bisceglia et al. (2009)
9q22.2	Southern Italian	25/AD	136 (77)	382 Highly polymorphic markers	1.61	2.10	*CTSL,PCSK5*	–	–	Bisceglia et al. (2009)
14q11.2	Southern Italian	25/AD	136 (77)	382 Highly polymorphic markers	2.09	2.62	*APEX1*	–	–	Bisceglia et al. (2009)
15q15.1	Southern Italian	25/AD	136 (77)	382 Highly polymorphic markers	1.74	2.32	–	–	–	Bisceglia et al. (2009)
16q23.1	Southern Italian	25/AD	136 (77)	382 Highly polymorphic markers	0.45	1.97	*CDK10*	–	–	Bisceglia et al. (2009)
18p11.31	Southern Italian	25/AD	136 (77)	382 Highly polymorphic markers	0.72	2.00	–	–	–	Bisceglia et al. (2009)
13q32	Ecuadorian	18/AD	143 (76)	763 Fluorescently labeled PCR primer pairs	4.1	3.2	*MBNL1, IPO5, FARP1, RNF113B, STK24, DOCK9, ZIC5, ZIC2*	*MBNL1, IPO5, FARP1, RNF113B, STK24, DOCK9, ZIC5, ZIC2* (Sanger sequencing)	*DOCK9* c.2262A>C DOCK9 c.720+43A>G IPO5 c.2377-132A>C STK24 c.1053+29G>C	Gajecka et al. (2009), Czugala et al. (2012), Karolak et al. (2016b)
13q34	Ecuadorian	18/AD	143 (76)	763 Fluorescently labeled PCR primer pairs	2.86	–	*COL4A1, COL4A2*	*COL4A1, COL4A2* (Sanger sequencing)	No mutations	Gajecka et al. (2009) Karolak et al. (2011)
14q24.3	White English, Iranian, Indian, Pakistani	6/AD	35 (23)	10,805 SNP markers	3.58	–	*VSX2*	*VSX2* (Sanger sequencing)	No mutations	Liskova et al. 2010
5q31	Ecuadorian	1/AD	8 (6)	811 Microsatellite markers	2.32	0.53	*PITX1, IL9, TGFBI*	*PITX1, IL9, TGFBI* (Sanger sequencing)	No mutations	Rosenfeld et al. (2011), Karolak et al. (2016b)
11p15.5-p15.4	Ecuadorian	1/AD	20 (7)	811 Microsatellite markers	–	1.66	–	–	–	Nowak et al. (2012)
2q13-q14.3	Ecuadorian	1/AD	21 (9)	811 Microsatellite markers	–	2.39	*IL1A, IL1B, IL1RN*	*IL1A, IL1B, IL1RN* (Sanger sequencing)	*IL1RN* c.214+242C>T	Nowak et al. (2013)
20p13-p12.2	Ecuadorian	1/AD	21 (9)	811 Microsatellite markers	–	2.40	*SLC4A11*	*SLC4A11* (Sanger sequencing)	*SLC4A11* c.2558+149_2558+203del54	Nowak et al. (2013)
5q15-q21.1	Caucasian	1/AD	27 (14)	525,000 SNP markers	2.49	–	–	–	–	Bykhovskaya et al. (2016)

^a^Keratoconus with Leber congenital amaurosis

^b^AR: Autosomal recessive mode of inheritance

^c^AD: Autosomal dominant mode of inheritance

^d^Keratoconus with cataract

^e^Digenic approach

Among the previously reported chromosomal regions, only loci at 5q have been replicated (Tang et al. 2005; Li et al. 2006; Bisceglia et al. 2009; Rosenfeld et al. 2011; Bykhovskaya et al. 2016). One of them is 5q21.2 identified in Caucasians (Tang et al. 2005) and confirmed in Caucasians originated from Italy (Bykhovskaya et al. 2016). The second locus is 5q31.1-q35.3 identified in Ecuadorian KTCN family (Rosenfeld et al. 2011; Karolak et al. 2017) that overlapped 5q31 and 5q32-q33 loci, previously reported in Caucasian/Hispanic and the Southern Italian population (Li et al. 2006; Bisceglia et al. 2009). The remaining KTCN susceptibility loci were representative for specific populations, small numbers of families, or most frequently, for single families only. This locus heterogeneity makes it difficult to identify genes unambiguously influencing the KTCN.

The finding of putative KTCN genes is additionally complicated by the fact that chromosomal intervals mapped by linkage analyses are usually large in size (up to several megabases) and contain numerous candidate genes to be further evaluated. Mutation screening of all putative genes from these regions requires substantial additional effort; thus, post-linkage analysis is often narrowed to the sequencing of a few functional genes localized in the genetic interval between markers with the maximum LOD. However, functional and positional candidate genes often do not contain variants related to the disease phenotype.

*COL8A1* (collagen type VIII, alpha 1) gene, localized at 3p14-q13 KTCN is a good example for that (Brancati et al. 2004). Collagens play an important role in corneal stroma organization. *Col8a1* and *Col8a2* knock-out mice demonstrated that type VIII collagen is required for normal anterior eye development, particularly the formation of a corneal stroma (Hopfer et al. 2005). Keeping this in mind, *COL8A1* gene seemed to be the best functional candidate genes at 3p14-q13 KTCN locus. Interestingly, molecular screening of coding regions of *COL8A1* has not revealed any potentially pathogenic variant (Brancati et al. 2004; Aldave et al. 2007). Similar situation was with *COL4A1* and *COL4A2*. Since it was known that type IV collagen may be involved in KTCN (Stachs et al. 2004), these two genes localized at 13q34 linkage region were the first choice targets for molecular screening. Again, the analysis showed no pathogenic variants (Karolak et al. 2011).

An another issue complicating finding of putative genes in linkage regions is the fact that promising candidate genes may be localized in close proximity to the highest linkage peak and not exactly in the peak. In an Ecuadorian family, variants fully segregating with the KTCN phenotype were identified in the *IPO5* (importin 5), *STK24* (serine/threonine kinase 24), and *DOCK9* (dedicator of cytokinesis 9) genes (Table 1). These genes are mapped in distance (0.12 Mbp, 0.43 kbp, and 0.34 Mbp, respectively) to *FARP1* (FERM, RhoGEF, and pleckstrin domain-containing protein 1), for which the maximum LOD score (4.1 in multipoint parametric linkage and 3.2 for multipoint non-parametric linkage) has been obtained. Interestingly, molecular screening of *FARP1* has revealed no pathogenic variants in the affected members of the KTCN-014 family (Gajecka et al. 2009; Czugala et al. 2012). Causative genes might also be mapped in genomic regions with low linkage signals (Nowak et al. 2013). Screening of *IL1RN* (interleukin 1 receptor antagonist) and *SLC4A11* (solute carrier family 4 member 11) at 2q13-q14.3 and 20p13-p12.2 (LOD scores of 2.395 and 2.409, respectively) has revealed variants that were observed significantly more frequently in family members with KTCN (Nowak et al. 2013).

Apart from the difficulties in selecting candidate genes for further analysis, another limitation of linkage studies is the need to ascertain a number of multigenerational families with many affected and unaffected individuals. Although some of the KTCN loci have been identified using linkage analysis of small KTCN families, no causative variants have been found in genes from those loci (Burdon and Vincent 2013). Moreover, as indicated above, some patients may have a *forme fruste* or early form of the disease that could not be properly recognized. Thus, some affected individuals might be classified as unaffected in the family pedigree, causing difficulties or errors in genotype-phenotype correlations during linkage analyses.

## Relationship between sequence variation and KTCN

An association analysis is the other approach implemented in KTCN genomic studies. One of the possible applications of this type of study is the assessment of the role of particular genetic variants in the development of KTCN. This method was used by Kim and colleagues to investigate the association between KTCN and variants in the *IL1B* (interleukin 1 beta) promoter gene, which may be involved in inflammatory processes in KTCN (Kim et al. 2008). The first remark about inflammatory process in KTCN eyes was reported in 1991. Fabre and co-workers showed that corneal fibroblasts presented fourfold more IL-1-binding sites than normal fibroblasts, suggesting that these corneal fibroblasts could have increased sensitivity to IL-1 compared with normal corneal fibroblasts (Fabre et al. 1991). IL-1 is critical for the inflammatory process and could participate in triggering apoptosis of the corneal cells, which might lead to the development of KTCN (Wilson et al. 1996). Kim and co-workers found that c.-31T>C and c.-511C>T genetic variants in the *IL1B* were associated with a significantly increased risk of KTCN in Korean patients (Kim et al. 2008). Subsequent studies have also shown alterations in the genes involved in IL-1-dependent processes, including *IL1RN* gene (Nowak et al. 2013). Association of IL-1-related single-nucleotide variants (SNVs) with KTCN may suggest that some inflammatory events are responsible for KTCN (Nowak et al. 2013).

The second application of association analysis, genome-wide association study (GWAS), is used to identify the common sequence variation that contributes to a disease risk. This type of study is performed in case–control cohorts with hundreds or thousands individuals and allows for genome-wide analyses due to microarray-based SNP genotyping (Manolio 2010).

The first GWAS results for KTCN were reported in 2011 by Burdon and co-workers. Analyses performed in cohorts from USA, Australia, and Northern Ireland have led to the identification of a positive association of KTCN with two SNPs, rs1014091 and rs3735520. These SNPs are localized in both the promoter and upstream region of the *HGF* (hepatocyte growth factor) gene, which was previously reported as a high myopia gene in a Han Chinese population (Han et al. 2006; Burdon et al. 2011) (Table 2). A statistically significant association between *HGF* and KTCN was also found in Australian patients showing that this locus might be relevant and contributed to disease susceptibility (Sahebjada et al. 2014). The HGF contains binding sites for the cytokine IL-6, which may suggest that *HGF*, like *IL1A*, *IL1B,* and *IL1RN*, is involved in KTCN through inflammatory pathways (Burdon et al. 2011).

**Table 2:** Genome-wide significant association with keratoconus (data shown in chronological order)

Gene	Population	Discovery/replication cohort case/control	SNPs	*p* value	Meta *p* value	Genotyping methods	References
(a) Genes identified in GWAS as associated with keratoconus							
*HGF*	Australian Northern Ireland	97/216	rs3735520 rs17501108/rs1014091	0.002 0.0002	9.9 × 10^−7^ 9.9 × 10^−5^	IluminaHumanHap 1 M	Burdon et al. (2011)
*HGF*	Caucasian (US)	222/3324	rs3735520 rs17501108/rs1014091	6.1 × 10^−7^ 0.018	9.9 × 10^−7^ 9.9 × 10^−5^	Illumina HumanCNV370-Quad BeadChip Illumina iSelect Infinium	Burdon et al. (2011)
*RAB3GAP1*	Caucasian (US)	222/3324	rs4954218	2.6 × 10^−4^	1.6 × 10^−7^	Illumina HumanCNV370-Quad BeadChip Illumina iSelect Infinium	Li et al. (2012)
*LOX*	Caucasian (US)	222/3324	rs10519694 rs2956540	2.3 × 10^−3^ 7 × 10^−3^	4.0 × 10^−5^ 7.7 × 10^−4^	Illumina HumanCNV370-Quad BeadChip Illumina iSelect Infinium	Bykhovskaya et al. (2012)
*FOXO1*	Australian/Caucasian (US)	874/6085	rs2721051	2.7 × 10^−10^	–	Sequenom Autoflex MassArray Illumina HumanHap610-Quad BeadChip Illumina HumanCNV370-Quad BeadChip	Lu et al. (2013)
*FNDC3B*	Australian/Caucasian (US)	874/6085	rs4894535	4.9 × 10^−9^	–	Sequenom Autoflex MassArray Illumina HumanHap610-Quad BeadChip Illumina HumanCNV370-Quad BeadChip	Lu et al. (2013)
*RXRA*-*COL5A1*	Australian/Caucasian (US)	874/6085	rs1536482	2.6 × 10^−7^	-	Sequenom Autoflex MassArray Illumina HumanHap610-Quad BeadChip Illumina HumanCNV370-Quad BeadChip	Lu et al. (2013)
*MPDZ*-*NF1B*	Australian/Caucasian (US)	874/6085	rs1324183	5.2 × 10^−6^	–	Sequenom Autoflex MassArray Illumina HumanHap610-Quad BeadChip Illumina HumanCNV370-Quad BeadChip	Lu et al. (2013)
*COL5A1*	Australian/Caucasian (US)	874/6085	rs7044529	8 × 10^−6^	–	Sequenom Autoflex MassArray Illumina HumanHap610-Quad BeadChip Illumina HumanCNV370-Quad BeadChip	Lu et al. (2013)
*BANP*-*ZNF469*	Australian/Caucasian (US)	874/6085	rs9938149	1.9 × 10^−4^	–	Sequenom Autoflex MassArray Illumina HumanHap610-Quad BeadChip Illumina HumanCNV370-Quad BeadChip	Lu et al. (2013)
*COL5A1*	Caucasian (US)	222/3324	rs1536482 rs7044529	6.5 × 10^−3^ 7.4 × 10^−3^	1.5 × 10^−4^ 2.9 × 10^−3^	Illumina HumanCNV370-Quad BeadChip Illumina iSelect Infinium	Li et al. (2013a)
(b) Genes identified in GWAS as associated with keratoconus and replicated in independent studies							
*RAB3GAP1*	Australian Caucasian	524/2761	rs4954218	9.26 × 10^−9^	–	Sequenom Autoflex MassArray Illumina HumanHap610-Quad BeadChip	Bae et al. (2013)
*MPDZ*-*NF1B*	Australian Caucasian	157/673	rs1324183	0.001	–	Sequenom Autoflex MassArray	Sahebjada et al. (2013)
*BANP*-*ZNF469*	Australian Caucasian	157/673	rs9938149	0.010	–	Sequenom Autoflex MassArray	Sahebjada et al. (2013)
*HGF*	Australian Caucasian	157/673	rs2286194	1.1 × 10^−3^	–	Sequenom Autoflex MassArray	Sahebjada et al. (2014)
*LOX*	Iranian	112/150	rs1800449	0.012	–	Allele-specific PCR	Hasanian-Langroudi et al. (2015)

While some sequence variants in genes involved in IL-dependent processes were identified, the role of inflammatory events in KTCN remains controversial. There is no evidence of systemic inflammation, and there were no statistically significant differences in the level of inflammatory cytokines in serum between normal and KTCN individuals (Jun et al. 2011). Due to the absence of typical clinical signs of inflammation in KTCN patients, KTCN was traditionally considered as a non-inflammatory corneal disorder. However, recent proteomic studies have provided a wide spectrum of evidence of local inflammation in KTCN patients, which may support the hypothesis by McMonnies (2015) that it may be appropriate to classify KTCN as a quasi-inflammatory disease. (McMonnies 2015). Galvis and co-workers also suggested that KTCN could be, at least in part, an inflammatory condition (Galvis et al. 2015).

The other GWAS has revealed the association between rs4954218 SNP, mapped upstream of the *RAB3GAP1* gene (RAB3GTPase-activating protein) and KTCN in a Caucasian cohort from the USA (Li et al. 2012). These findings were replicated in Australian Caucasian KTCN cases, providing additional evidence of significant association of rs4954218 with KTCN (Bae et al. 2013). Evidence of a genetic association was also found between KTCN and rs10519694 and rs2956540 SNPs located in the *LOX* (lysil oxidase) gene, encoding an enzyme responsible for collagen cross-linking in different tissues, including the cornea (Bykhovskaya et al. 2012). This association was primarily identified in two independent case–control panels and in Caucasian and Hispanic families with KTCN (Bykhovskaya et al. 2012), followed by a replication in Iranian patients (Hasanian-Langroudi et al. 2015). A most recent GWAS has identified six loci associated with central corneal thickness and KTCN-containing polymorphisms in the *FNDC3B*, *MPDZ*-*NF1B*, *RXRA*-*COL5A1*, *COL5A1*, *FOXO1*, and *BANP*-*ZNF469* genes (Lu et al. 2013). Among them, *MPDZ*-*NF1B* and *BANP*-*ZNF469* association was confirmed by an independent study, performed in an Australian Caucasian cohort (Sahebjada et al. 2013). A detailed description of all loci reported to be associated with KTCN is presented in Table 2. The case–control association analyses between KTCN in a Saudi Arabian cohort and particular SNPs from the genomic regions of *FNDC3B*, *MPDZ*-*NF1B*, *RXRA*-*COL5A1*, *FOXO1*, *LCN12*-*PTGDS*, and *BANP*-*ZNF469* did not reach the level of a statistically significant association (Abu-Amero et al. 2015a). Genotyping of the SNPs in *RAB3GAP1*, *FNDC3B*, *LOX*, *HGF*, *MPDZ*-*NF1B*, *RXRA*-*COL5A1*, *COL5A1*, *FOXO1*, and *BANP*-*ZNF469* in the Han Chinese KTCN patients revealed that only the SNP located in *MPDZ*-*NF1B* was associated with an increased risk of KTCN in this population. No significant difference was observed between KTCN and controls in other SNPs including rs9938149 from *BANP*-*ZNF469* genomic region (Hao et al. 2015).

Despite computational and experimental developments, the interpretation of GWAS data is still a great challenge. Discovery of a causative variant is difficult especially if the associated sequence variant is located in a non-coding region or in a distance from known genes. These inter- and intra-genic regions of human genomes may contain regulatory elements, but a little is known regarding how particular variants affect their functions and how this influences the phenotype. Therefore, the statistically significant association of a particular SNP with a disease does not prove that this variant is actually causative for the disorder and further studies are required to assess its role in disease development.

Another limitation of GWAS analyses is the size of the study group. For complex traits, such as KTCN, meaningful results can be obtained with several hundreds or thousands of individuals with a disease phenotype. In addition, poor phenotypic classification or inconsistent phenotype exams (using different inclusion criteria in various ascertainment places) in patients may lead to false positive associations. Moreover, significant loci often explain only a small proportion of the phenotypic variation, suggesting that rare variants may underlie the genetic causes of the disease. As GWAS recognizes only common risk alleles, the locus associated with GWAS signals may be far larger than assumed. Thus, a good solution for revealing rare variants would be to extend the sequencing region to at least a few additional megabases around the GWAS signal (Dickson et al. 2010). Rare alleles may also be discovered through whole-exome sequencing (WES) or whole-genome sequencing (WGS) analyses, which will be discussed below.

## Sanger sequencing mutational screening

For the last few decades, automated Sanger sequencing was the gold standard for human genome research. To date, numerous promising genes implicated in KTCN etiology have been analyzed using this tool (Table 3). Among them were *VSX1* (visual system homeobox 1) and *SOD1* (superoxide dismutase isoenzyme 1), the first two genes proposed as significant for KTCN development. The gene *VSX1* is mapped to chromosome 20p11.2 and encodes a vertebrate paired-like homeodomain transcription factor. It is expressed in vitro and in vivo in human keratocytes during their differentiation into myofibroblasts in response to wound healing (Barbaro et al. 2006). SOD1 is involved in oxidative stress-related processes, which may have a role in the etiology of KTCN (Udar et al. 2006). While the potentially disease-causing variants were initially identified in both genes (Héon et al. 2002; Udar et al. 2006), subsequent studies have not confirmed the original findings (Stabuc-Silih et al. 2010). As indicated in Table 3a, *VSX1* is the most evaluated gene in KTCN studies. To unambiguously determine the impact of *VSX1* on KTCN phenotype, the phenotype of corresponding mouse knock-out mutants is important. Although the *Vsx1* null mice were produced, their histological analyses did not show pathological alterations in the cornea (Chow et al. 2004). Considering the lack of KTCN in *Vsx1* knock-out mice and the absence of pathogenic variants in *VSX1* in general KTCN population, the role of *VSX1* in the pathogenesis of KTCN remains elusive.

**Table 3:** Candidate genes screened in keratoconus patients that were not indicated in linkage and genome-wide association studies (data shown in chronological order)

Studied population	Method	No. of affected individuals/no. of unaffected individuals (or no. of families)	References
(a) VSX1 as the well-studied gene in keratoconus			
Caucasian	PCR-SSCP, Sanger sequencing	63/277	Héon et al. (2002)
Italian	Sanger sequencing	80/125	Bisceglia et al. (2005)
Not described	Sanger sequencing	100/0	Aldave et al. (2006)
British, Indians, Pakistani, Africans, African-Caribbeans	Sanger sequencing	85 families	Liskova et al. (2007)
White population	ABI allelic discrimination technology, PCR-RFLP	77/71	Tang et al. (2008)
Ashkenazi Jewish	Sanger sequencing	1 family	Eran et al. (2008)
Korean	PCR-SSCP, Sanger sequencing	249/208	Mok et al. (2008)
Indian	Sanger sequencing	66/100	Paliwal et al. (2009)
Slovenian	Sanger sequencing	113/100	Stabuc-Silih et al. (2010)
European	Sanger sequencing	66/100	Dash et al. (2010)
Indian	Sanger sequencing	50/50	Tanwar et al. (2010)
Indian	Sanger sequencing	2/4	Paliwal et al. (2011)
Saudi Arabia	Sanger sequencing	55/50	Abu-Amero et al. (2011)
Iranian	Sanger sequencing, ARMS-PCR	112/52	Saee-Rad et al. (2011)
Korean	Sanger sequencing	53/100	Jeoung et al. (2012)
South Indian	Sanger sequencing	170/108	Verma et al. (2013)
Caucasian, Polynesian, Indian	Sanger sequencing	47/10	Vincent et al. (2013)
Iranian	PCR-SSCP, Sanger sequencing	50/50	Dehkordi et al. (2013)
Han Chinese	Sequenom Mass Array genotyping	97/101	Wang et al. (2013)
Greek	Sanger sequencing	33/78	Moschos et al. (2015)
Indian	Sanger sequencing	8 families	Shetty et al. (2015)
Polish	Sanger sequencing	42/50	Karolak et al. (2016b)

Gene	Studied population	Method	Case/control (or no. of families)	References
(b) Other genes analyzed in keratoconus patients				
*CAST*	Multiethnic	TaqMan genotyping	304/518	Li et al. (2013b)
*COL4A3*	Slovenian	SSCA, Sanger sequencing	104/157	Stabuc-Silih et al. (2009)
	Han Chinese	Sequenom Mass Array genotyping	97/101	Wang et al. (2013)
	Greek	Sanger sequencing	45/78	Kokolakis et al. (2014)
*COL4A4*	Slovenian	SSCA, Sanger sequencing	104/157	Stabuc-Silih et al. (2009)
	Han Chinese	Sequenom Mass Array genotyping	97/101	Wang et al. (2013)
	Greek	Sanger sequencing	45/78	Kokolakis et al. (2014)
	Iranian	Tetra-ARMS-PCR	112/150	Saravani et al. (2015)
*FAS*	Polish	HRM genotyping, TaqMan genotyping	264/300	Synowiec et al. (2014)
*FASLG*	Polish	HRM genotyping, TaqMan genotyping	264/300	Synowiec et al. (2014)
*FEN1*	Polish	PCR-RFLP	279/322	Wojcik et al. (2014b)
*IL1A*	Han Chinese	Sequenom Mass Array genotyping	97/101	Wang et al. (2013)
*IL1B*	Korean	PCR-RFLP, Sanger sequencing	100/100	Kim et al. (2008)
	Han Chinese	Sequenom Mass Array genotyping	97/101	Wang et al. (2013)
	Japanese	TaqMan genotyping	169/390	Mikami et al. (2013)
	Turkish	PCR-RFLP	121/121	Palamar et al. (2014)
*ILRN*	Turkish	PCR-RFLP	121/121	Palamar et al. (2014)
*LIG3*	Polish	genotyping	283/300	Synowiec et al. (2015)
*RAD51*	Polish	PCR-RFLP	100/150	Synowiec et al. (2013)
*SOD1*	Multiethnic	Sanger sequencing	15 Probands/156	Udar et al. (2006)
	Slovenian	Sanger sequencing	113/100	Stabuc-Silih et al. (2010)
	Iranian	Sanger sequencing, ARMS-PCR	112/52	Saee-Rad et al. (2011)
	Greek	Sanger sequencing	33/78	Moschos et al. (2015)
	Saudi Arabia	Sanger sequencing	55/100	Al-Muammar et al. (2015)
*TF*	Polish	PCR-RFLP	216/228	Wójcik et al. (2013)
*TGFBI*	American	Sanger sequencing	15 Probands	Udar et al. (2004)
	Chinese	Sanger sequencing	30/30	Guan et al. (2012)

Although the first two candidate genes, *VSX1* and *SOD1*, were identified, many other genes have been assessed as causative for KTCN. However, Sanger sequencing of numerous genes with functional or positional relevance for KTCN, including *VSX2* (visual system homeobox 2), *CTSH* (cathepsin H), *CRABP1* (cellular retinoic acid binding protein 1), and *RASGRF1* (RAS protein-specific guanine nucleotide-releasing factor 1), has revealed no significant variants in KTCN patients (Table 3b) (Hughes et al. 2003; Liskova et al. 2010). The absence of causative KTCN variants in the coding regions of these genes indicates that rare coding variants are not involved in KTCN pathogenesis. While intronic variants and other regulatory elements were not assessed in those studies, the exact genotype–phenotype correlations still remain unclear.

Sanger sequencing has led to the identification of a possible KTCN-related variant, c.2262A>C in *DOCK9*. *DOCK9* encodes a protein that possesses guanosine triphosphate/guanosine diphosphate exchange factor activity and specifically activates G-protein, CDC42, which is involved in intracellular signaling networks (Kwofie and Skowronski 2008). The identified c.2262A>C substitution leads to the replacement of glutamine by histidine at the highly conserved #754 position of the protein encoded by *DOCK9,* indicating that this gene might contribute to the KTCN phenotype in the analyzed Ecuadorian family (Czugala et al. 2012). Recently, the effect of c.2262A>C substitution in exon 20 of *DOCK9* was assessed in vitro. It was demonstrated that this particular variant has led to a splicing defect, resulting in the changed ratio between two DOCK9 isoforms: a wild-type transcript and a transcript without exon 20 (Karolak et al. 2015).

Interestingly, c.2262A>C in *DOCK9* together with other sequence variants localized in intronic regions of *DOCK9* (c.720+43A>G), *IPO5* (c.2377-132A>C), and *STK24* (c.1053+29G>C) formed a disease-related haplotype that was carried by all affected individuals in the Ecuadorian KTCN family (Czugala et al. 2012). As mentioned above, the non-coding regions of genes contain many regulatory elements and intronic alterations, including single-nucleotide changes, which may trigger a deleterious effect on pre-messenger RNA splicing (Lomelin et al. 2010). Thus, it might be hypothesized that identification of additional three sequence variants in the 13q32 KTCN linked region could be non-accidental.

The *ZNF469* (zinc finger protein 469) is another candidate gene screened in KTCN patients. The pathogenic mutations in *ZNF469* were originally reported in patients with brittle cornea syndrome, a condition characterized by an extremely thin cornea that tends to rupture (Abu et al. 2008). It was also showed that variants in *ZNF469* might contribute to central corneal thickness (CCT), which is abnormal in a wide variety of corneal diseases, including KTCN or corneal dystrophies (Lu et al. 2010, 2013; Vitart et al. 2010). The initial findings about an association between CCT and rs12447690 and rs9938149, mapped in the region upstream to *ZNF469*, were reported in patients from Australia and the United Kingdom, and then were replicated in Croatian and Scottish, Indian and Malays, Caucasians, and in Latinos (Lu et al. 2010; Vitart et al. 2010; Vithana et al. 2011; Hoehn et al. 2012; Gao et al. 2013). The independent replication of GWAS results suggests the possible involvement of these common SNPs in CCT variation in the general population. As was indicated above, a recent GWAS for CCT and KTCN performed in the European and Asian populations has identified six loci that were strongly associated with a KTCN risk, including the locus upstream to *ZNF469* (Lu et al. 2013).

The molecular screening of *ZNF469* gene identified a significant enrichment of potentially pathogenic heterozygous alleles in *ZNF469* in 12.5% of European KTCN patients (Lechner et al. 2014) and in 23.0% of Polynesian patients (Vincent et al. 2014). To determine whether genetic variants in *ZNF469* truly contribute to the disease, the full-length sequence of this gene was analyzed in unrelated Polish patients with isolated KTCN. Interestingly, sequencing revealed that average number of non-synonymous variants per one individual was comparable for KTCN and healthy Polish individuals and was 16.31 and 18.0, respectively (Karolak et al. 2016b). Based on the previous findings, it appears that the role of heterozygous variants in *ZNF469* in KTCN patients should be further evaluated. The high prevalence of *ZNF469* variants noted in KTCN individuals is typical for a common genetic variation observed in the general population. Moreover, the high phenotypic heterogeneity demonstrated by *ZNF469* gene mutations may indicate that similar processes are involved in diseases characterized by corneal thinning, in general, rather than only in KTCN (Davidson et al. 2015; Karolak et al. 2016a).

## Mitochondrial studies

In addition to the numerous sequencing studies focused on genomic DNA, a few studies have also been conducted on the level of mitochondrial DNA to identify further genetic aspects of KTCN. An analysis of mitochondrial complex I genes (*ND1*-*ND6*) in 20 Indian KTCN patients has revealed 84 variants, including two novel frameshift variants (Pathak et al. 2011). An investigation of full mitochondrial DNA extracted from blood samples of KTCN cases from Saudi Arabia has revealed mitochondrial DNA variants in 38.5% of KTCN patients. Among identified changes, only one non-synonymous variant (m.4218T>A in *ND1*) was heteroplasmic, whereas the remaining nine were homoplasmic (Abu-Amero et al. 2014). Homoplasmic mutations may lead to several diseases, but also heteroplasmic changes might be pathogenic, if the ratio between wild-type and altered mtDNA exceeds a given threshold value. The high level of heteroplasmy (74%) in studies performed by Abu-Amero and colleagues suggests that the identified change in *ND1* might be involved in KTCN (Abu-Amero et al. 2014). However, further studies are needed to determine the role of heteroplasmic changes in KTCN. As it is known that the traditional PCR followed by direct Sanger sequencing is insufficient to detect heteroplasmy, and it also lacks the necessary sensitivity to identify low levels of mosaicism (Jamuar et al. 2014; Gajecka 2016), techniques other than Sanger sequencing should be applied to assess heteroplasmy in KTCN.

Analysis of mitochondrial DNA gives insight into oxidative damage in KTCN. Oxidative stress is a consequence of the accumulation of reactive oxygen species (ROS) in different compartments of human body, including cornea, which cause a toxic effect on a number of cellular components, such as proteins, nucleic acids, and membrane phospholipids. ROS may be induced by UV light, and since the cornea is constantly exposed to the UV radiation, it is particularly vulnerable to the oxidative stress and damage from ROS (Buddi et al. 2002). The healthy cornea has several mechanisms involved in the minimization of ROS damaging effects, including both enzymatic activities and non-enzymatic pathways, which are altered in KTCN (Gondhowiardjo and van Haeringen 1993). It has been shown that besides the mitochondrial DNA damage, the level of antioxidants was decreased in KTCN corneas. Moreover, the level of oxidative stress markers was elevated, which suggested that mechanisms protecting against oxidative stress and cell degradation might be altered in this disease (Arnal et al. 2011; Wojcik et al. 2014a; Toprak et al. 2014). It is unclear whether oxidative cell degradation is a primary process in the disease development or it is a secondary effect.

## Next-generation sequencing as a tool for high-throughput data generation

As mentioned above, sequencing of numerous candidate genes is time-consuming and labor intensive and thus may be not effective in a large-scale study. These limitations can be reduced using next-generation sequencing (NGS). Current NGS technologies offer different methods for template preparation, sequencing, visualizing, genome alignment, and assembly. Still, each NGS strategy allows for direct and complete DNA sequencing in a high-throughput manner, as well as the production of an enormous volume of low-cost reads per instrument run (Metzker 2010). Since 2005, NGS platforms have become widely available, giving an impressive range of applications. The general DNA sequencing platforms offer WGS and targeted resequencing (Koboldt et al. 2013).

Targeted resequencing allows for focusing on all genomic regions of interest in one experiment. One of the strategies of targeted sequencing is whole-exome sequencing (WES). WES is a promising tool that enables selective sequencing of nearly all protein-coding sequences. Unlike the gold standard—exon-by-exon analysis by conventional Sanger sequencing—WES is time- and cost-effective and more suitable for the precise determination of rare and unique coding sequence variants in an individual exome. The literature data provide many examples of the efficacy of WES in variants identification in both rare and common complex disorders, including ocular diseases. Rare coding sequence variants have been found in patients with the sporadic and the familial form of retinitis pigmentosa (Jinda et al. 2014; Villanueva et al. 2014) and retinal dystrophy (Ortube et al. 2014). WES studies have also been successful in identifying mutations in other ophthalmic diseases, including high myopia (Zhao et al. 2013), cone-rod dystrophy (Huang et al. 2013), and corneal intraepithelial dyskeratosis (Soler et al. 2013), while in the literature, there is only one report about WES in KTCN research (Karolak et al. 2017). Also, targeted resequencing of a subset of the human genome was used by Hughes and colleagues in a selective screen of all genes within the 5.5 Mb region of chromosome 15q22-q25, which was previously reported as linked with KTCN in a Northern Irish family. That search resulted in the detection of c.57 C>T variant in the seed region of the *miR*-*184* gene in a family with severe KTCN combined with the early onset anterior polar cataract (Hughes et al. 2003, 2011).

The variants in *miR*-*184* were also identified in four subsequent studies performed in two Chinese patients with isolated KTCN; in patients with a syndrome characterized by endothelial dystrophy, iris hypoplasia, congenital cataract, and stromal thinning (EDICT); in members of family with congenital cataracts and corneal abnormalities including KTCN, and in Greek sporadic KTCN patients (Iliff et al. 2012; Lechner et al. 2013; Bykhovskaya et al. 2015; Moschos et al. 2016). In contrast, latest studies performed in Iranian and Saudi Arabian KTCN patients revealed no significant *miR*-*184* variants, which may suggest that alterations in *miR*-*184* are a rare cause of KTCN alone (Abu-Amero et al. 2015b; Farzadfard et al. 2016).

WES studies are restricted to determining the variants in protein-coding sequences, which constitute approximately only 1% of the whole human genome. Thus, many potentially important variants localized in the remaining parts of the genome would be missed. Other limitations of WES include both technical and post-sequencing issues. Some difficulties may result from insufficient sequencing coverage or inappropriate algorithms used for variant calling and annotation, especially in case of variants from multi-allelic sites. Despite the expanded databases and sophisticated workflows for data processing, both the narrowing of a large amount of initial data to a manageable number of variants and the filtering of candidate variants without a loss of possible causative ones are still challenging. While trio analysis (patient and both parents are included) in search of de novo mutations is quite simple, looking for hereditable mutations in large families with a complex disease, such as KTCN, could be problematic and requires additional Sanger-segregation analyses. Despite these limitations, WES is a promising tool to study genetic causes of many disorders. The increasing interest in new molecular techniques and easier access to the necessary equipment leads us to believe that this approach will be more frequently implemented in KTCN research in the near future.

As we indicated above, WES allows for analysis of the coding region of genes only. Since many DNA variants associated with diseases lie outside the coding regions of genomic DNA, WGS, which allows for the characterization of entire genomes of any size, has become a promising and more appropriate solution for research looking for the unidentified genetic causes of diseases. However, while the newest machines can process whole human genomes for only $1000 each, WGS is not routinely used in research laboratories. An expanded computational infrastructure is required in processing and interpreting data sets of full genomes. Literature data showed that WGS may be successfully implemented in the identification of pathogenic DNA variants in genetically heterogeneous diseases, including autism spectrum disorder and the autosomal recessive retinitis pigmentosa (Jiang et al. 2013; Nishiguchi et al. 2013). Since locus and allelic heterogeneity has been observed in KTCN studies, it is reasonable to assume that WGS will be a useful method for assessing KTCN etiology.

## Epigenetic studies

There are many changes in gene function, which are not elucidated by variations in the DNA sequence but may be explained by epigenetic modifications. Epigenetic mechanisms include, i.e., DNA methylation, and regulation of gene expression by small and non-coding RNAs. As epigenetic changes may be associated with the development and progression in several diseases, and then the epigenomic studies need to be added to molecular-based investigations of human disorders.

Methylation status in KTCN was studied in *TIMP3* (tissue inhibitor of metalloproteinase 3) promoter. It was shown that overexpression of *TIMP3* might induce apoptosis in corneal stromal cell cultures leading to KTCN (Matthews et al. 2007). Changes in DNA sequence of *TIMP3* were not identified suggesting that alteration of *TIMP3* expression might be due to epigenetic factors (De Bonis et al. 2011). However, methylation-specific quantitative PCR performed in three keratoconic and three healthy corneal tissues excluded promoter methylation in the *TIMP3* (De Bonis et al. 2011). Large-scale epigenetic studies have never been implemented in KTCN research.

## Challenges and outlook

- One of the issues limiting KTCN studies is a problem with the collection of the appropriate tissue for analyses. While blood samples are usually not difficult to collect from all studied individuals, the collection of whole corneal tissues or their fragment is limited to patients with corneal transplantation. Moreover, it is not possible to obtain the healthy cornea from living donors for comparative analyses. Therefore, the corneal tissues are often derived from non-KTCN individuals with other corneal events, who were referred for corneal transplantation.
- A good model for KTCN research could be cell lines derived from different parts of human corneas, i.e., corneal epithelial or endothelial cell lines, and corneal fibroblasts. However, the establishment and cultivation of cell lines are difficult and often fail. In addition, corneal cell lines as other cell lines derived from different cell types are not well characterized, have a limited life span, and may not reflect the properties of in vivo cells.
- There is sufficient data to suggest that KTCN has a strong genetic component. Based on genetic studies, it has become clear that KTCN etiology might involve more than one causing factor and the traditional molecular techniques may not be sufficient for identifying all elements involved in KTCN. The rapid advances of high-throughput molecular technologies have led to the development of innovative approaches allowing relationship analyses between disease phenotype and variations in the human genome. With increased using of next-generation sequencing technologies, it may be possible to identify genetic variants underlying the KTCN etiology.
